# Comparing the efficacy of sinus irrigation with traditional Caldwell–Luc procedure following odontogenic cyst surgery involving the maxillary sinus

**DOI:** 10.1038/s41598-021-97477-z

**Published:** 2021-09-13

**Authors:** Niu Gang, Chen JiaMin, Wu Ye, Xie FuPing, Liu HuanHuan, Lin LiSong

**Affiliations:** 1 Department of Oral and Maxillofacial Surgery, School and Hospital of Stomatology, Fujian Medical University, Fuzhou, China; 2 Department of Oral and Maxillofacial Surgery, The First Affiliated Hospital of Fujian Medical University, Fuzhou, China

**Keywords:** Head and neck cancer, Oral cancer

## Abstract

A large maxillary odontogenic cyst could intrude into the maxillary sinus. The traditional approach following surgery for such a cyst is the Caldwell–Luc procedure. However, the traditional CDL procedure is associated with more complications and damage of the sinus mucosa. The purpose of this study was to assess a new method with easier operation, which not only prevented postoperative infection but also caused less damage to the maxillary sinus mucosa. A large odontogenic cyst in the maxillary sinus of 40 patients was diagnosed through radiographic imaging and postoperative histopathology. Twenty patients were treated with maxillary sinus saline irrigation after surgery, while 20 patients underwent the traditional Caldwell–Luc procedure. The therapeutic efficacy was evaluated by clinical examination and radiographic imaging; the longest follow-up was 36 months. The postoperative reaction was evaluated. There was a statistically significant difference in facial swelling, visual analog scale (VAS) and temperature elevation between the 2 groups. Sinus irrigation following large odontogenic cyst surgery involving the maxillary sinus can serve as an alternative to standard CDL and has the advantages of fewer complications, reduced trauma, restoration of the mucosa and more satisfactory results.

## Introduction

Odontogenic cysts (OCs) are characterized as destructive maxillofacial bone lesions that develop from the odontogenic epithelium^1^. OCs can be divided into 2 main groups: inflammatory OCs, including radicular cysts (RCs), and developmental OCs, including dentigerous cysts and keratocysts^1^. Generally asymptomatic, OCs grow slowly. In maxillary bone, large OCs could intrude into the maxillary sinus and destroy the lateral wall of the maxillary sinus. Meanwhile, infection occurs in the form of chronic maxillary sinusitis (CMS)^2^.

Traditionally, cyst enucleation and the Caldwell–Luc (CDL) procedure are performed for OCs involving the maxillary sinus. However, complete stripping of the hyperplastic inflamed tissue and sinus lining often leads to sclerosis of the sinus walls and osteitis and damages the mucociliary function of the sinus lining^3^. At the end of these procedures, the cyst and maxillary sinus cavity are packed to prevent infection and hemorrhage, which may cause postoperative complications, including facial swelling, facial paresthesia, mild temperature elevation and recurrence of sinusitis^4^. Currently, functional endoscopic sinus surgery (FESS) and antral irrigation is known to be an effective treatment for otolaryngologist to treat with refractory chronic sinusitis and can improve patients’ symptoms and disease processes^5^. It allows not only minimal invasion but also a simultaneous treatment of hyperplastic, hypertrophic, or infected mucosa. Such treatment has proven to be less aggressive than CDL procedure^6^. This stimulated us to attempt a new technique for solution of this challenging problem.

Consequently, it is necessary to explore a new method with fewer complications that not only preserves the normal maxillary sinus mucosa but also reduces postoperative infection of the maxillary sinus. In this article, we present our experience with sinus irrigation following OC surgery. This technique is less invasive than traditional procedures, and the success rate and complications will be discussed.

## Patients and methods

This study followed a prospective, randomized controlled design and was approved by the Institutional Review Board of the School and Hospital of Stomatology, Fujian Medical University. All methods were performed in accordance with the relevant guidelines and regulations. All patients were informed of the surgical procedure, the potential benefits and risks. Informed consent was obtained for all patients. Between January 1, 2012, and January 31, 2018, a total of 40 patients with large OCs involving the maxillary sinus were enrolled in the study. Forty patients randomized to group A and group B. Preoperative the involved teeth were examined to confirm their vitality. In case they were non-vitals, the endodontic treatments were underwent before the surgery.

The inclusion criteria were as follows: (1) preoperative clinical examination diagnosed as odontogenic cyst of maxilla; (2) the preoperative otolaryngologist diagnosed unilateral maxillary sinusitis; (3) preoperative CT showed the cyst extending into the sinus with complete obliteration of the maxillary sinus; (4) the cyst was found to be adhered to the mucosa of the maxillary sinus during operation; (5) histopathological examination confirmed the diagnosis of OC postoperatively.

Group A was composed of 20 patients who were treated with maxillary sinus irrigation, and group B (control group) was composed of 20 patients who were treated with the traditional CDL approach.

**Figure 1:**
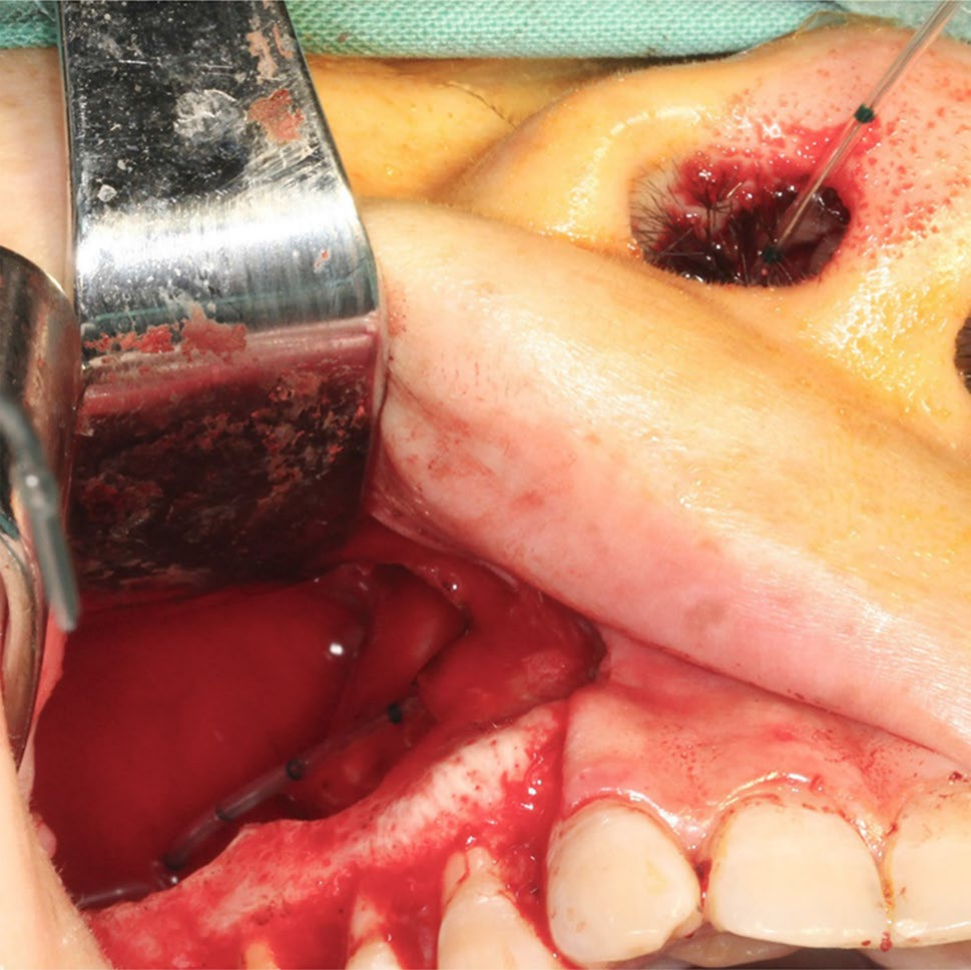
An epidural catheter was inserted through the nasal antrostomy into the cyst cavity.

**Figure 2:**
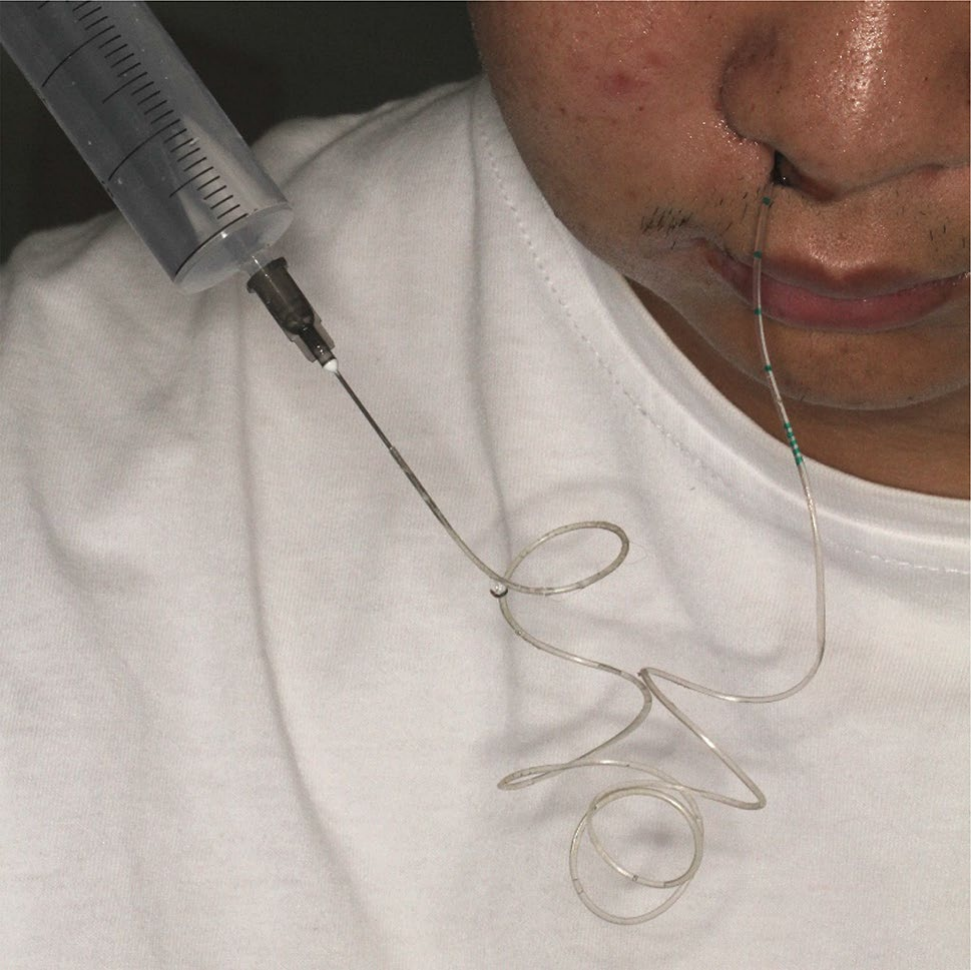
The maxillary sinuses were irrigated with saline through the epidural catheter.

All patients were treated under intravenous-inhalation anesthesia. The first surgical step was treatment of the cyst source. A gingival fold incision or intrasulcular incision with vertical releasing cuts was made in the area involving the cyst, and the cyst with adhesion sinus membrane which could not be separated was enucleated. In group A, the mucous membrane of the maxillary sinus was left as possible. After the completion of thorough cyst enucleation and hemostasis, inferior meatal antrostomy (IMA) was performed using an antrostomy knife. The entry point was chosen at the thinnest bony portion of the cyst wall according to the CT findings. An epidural catheter was inserted through the nasal antrostomy into the cyst cavity (Fig. 1). Then, the catheter was fixed near the base of the nose using sutures. In group B, the standard CDL procedure was performed, the diseased and inflammation sinus membranes were all enucleated and stripped down, the maxillary sinus cavity was packed for hemostasis. Finally, the intraoral incision was closed with absorbable sutures. After surgery, antibiotics (2000 mg of cefazolin intravenously 2 times daily for 3 days) were prescribed for all operated patients. The patients were instructed to take sinus-related precautions for 2 weeks. In group A, maxillary saline irrigation was performed as follows: the maxillary sinuses were irrigated with 100 mL of normal saline using a 20-mL injection syringe through the epidural catheter twice a day (Fig. 2). During saline irrigation, the patients lowered their head, and the fluid flowed out through the nasal passages. Patients treated with saline irrigation underwent removal of the epidural catheter 2 weeks after surgery. The postoperative variables as facial swelling, pain intensity and temperature elevation was evaluated.

## Results

The diameter of the cyst was not complied with the normal distribution so it represented by quartile method. In group A 11(55%) were males, 9(45%) were males, median age of patient was 37.74 (11) years, median diameter of the cyst was 44.35 mm. In group B 10(50%) were males, 10(50%) were males, median age of patient was 39.50 (10) years, median diameter of the cyst was 44.65 mm. There was no statistically significant difference in age, sex and diameter of cyst between the groups. All the patients in groups A and B were cured. The indications for the approaches in groups A and B are summarized in Table 1. Information regarding various parameters immediately postoperatively is presented in Tables 2 and 3. Facial swelling was recorded in all patients on postoperative day 2, including mild swelling (almost unnoticeable swelling in the infraorbital region) in group A (6 patients, 30%) and group B (2 patients, 10%), moderate swelling (noticeable swelling in the infraorbital region) in group A (12 patients, 60%) and group B (12 patients, 60%), and severe swelling (significant swelling in the infraorbital region extending to the periorbital region) in group A (2 patients, 10%) and group B (6 patients, 30%). There was a statistically significant difference in facial swelling between the 2 groups (Wilcoxon rank sum test, P < 0.05). The swelling usually decreased starting on the third day after surgery and resolved within 1 week.

**Table 1:** Indications for the approaches in group A and group B.

Indication	Group A	Group B
Radicular cyst	10	13
Dentigerous cyst	8	6
Keratocyst	2	1

**Table 2:** Immediate postoperative complications in group A.

Immediate postoperative complications	No. of patients	%
**Postoperative facial swelling**		
Mild (fullness sensation only)	6	30
Moderate (swelling without infraorbital involvement)	12	60
Severe (swelling with infraorbital involvement)	2	10
**Postoperative facial pain (visual analog scale, VAS)**		
Mild (VAS score, 1–3)	16	80
Moderate (VAS score, 4–6)	4	20
Severe (VAS score, 7–10)	0	0
Fever (> 38.0 °C)	1	5
Nasal obstruction	0	0
Hemorrhage	0	0

**Table 3:** Immediate postoperative complications in group B.

Immediate postoperative complications	No. of patients	%
**Postoperative facial swelling**		
Mild (fullness sensation only)	2	10
Moderate (swelling without infraorbital involvement)	12	60
Severe (swelling with infraorbital involvement)	6	30
**Postoperative facial pain (visual analog scale, VAS)**		
Mild (VAS score, 1–3)	10	50
Moderate (VAS score, 4–6)	6	30
Severe (VAS score, 7–10)	4	20
Fever (> 38.0 °C)	8	40
Nasal obstruction	0	0
Hemorrhage	0	0

The pain intensity was recorded in all patients on postoperative day 1 as assessed by the visual analog scale (VAS). For each patient, pain was recorded as follows: mild pain (VAS score, 1–2) occurred in 16 patients (80%) in group A and 10 patients (50%) in group B, moderate pain (VAS score, 3–4) occurred in 4 patients (20%) in group A and 6 patients (30%) in group B, and severe pain (VAS score, 5–6) occurred in 0 patients (0%) in group A and 4 patients (20%) in group B. Mean (SD) VAS was 2.50(1.43) and 4.15(2.78) in group A and group B respectively with a P value < 0.05 with statistically significant difference(T test).

On the second day after surgery, a mild temperature elevation (38.0 °C-39.0 °C) was noted in 8 patients (40%) in group B and 1 (5%) patient in group A. There was a statistically significant difference in temperature elevation between the 2 groups (Chi-squared test, P < 0.05) (Tables 1, 2).

**Figure 3:**
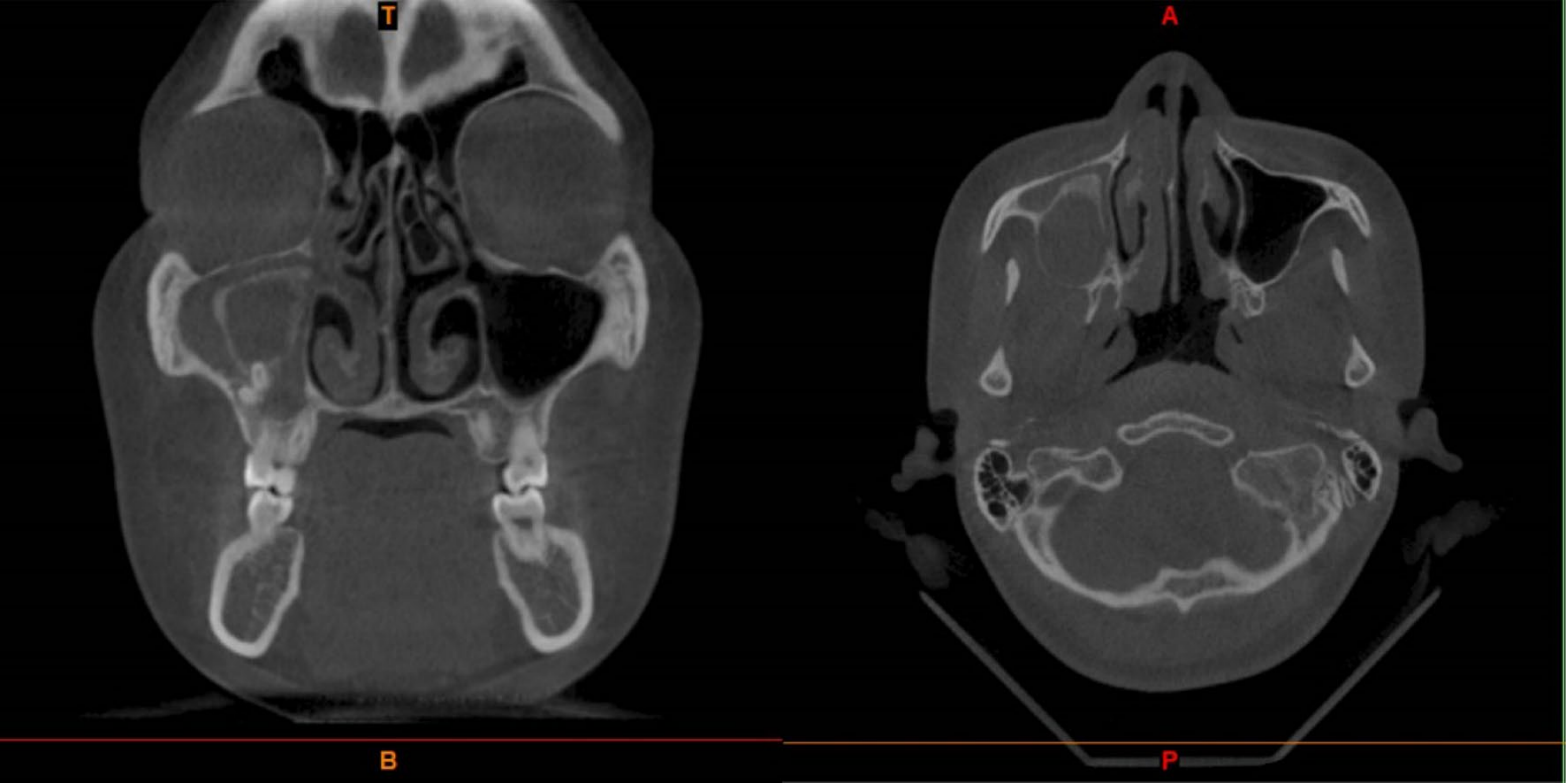
Preoperative Computed tomography showing large cyst in the right maxillary sinus with CMS.

**Figure 4:**
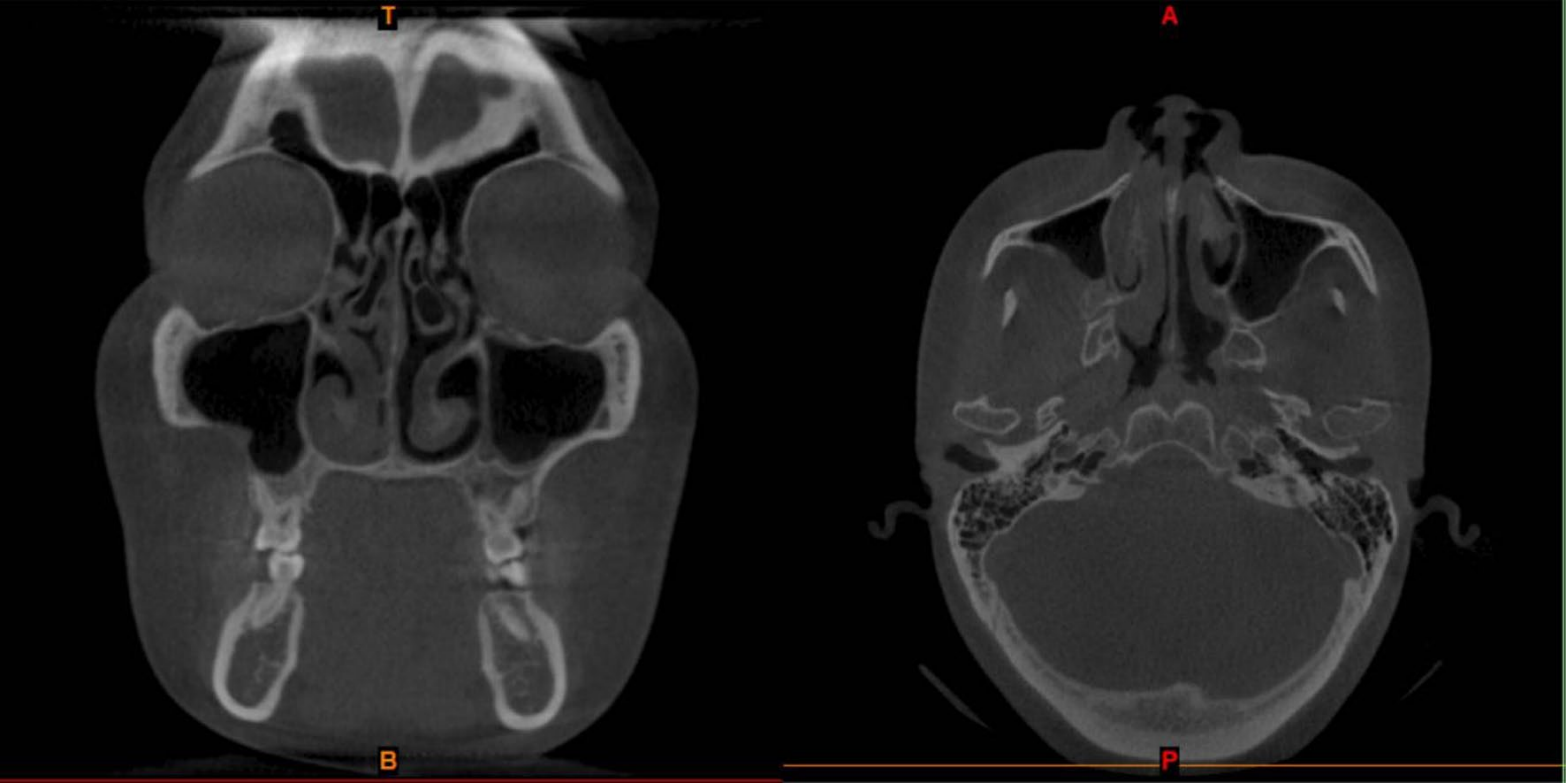
Post-operative CT scan showing a clear maxillary sinus at 12 weeks in the same patient (Figs. 1, 2, 3 and this figure is the same patient).

**Figure 5:**
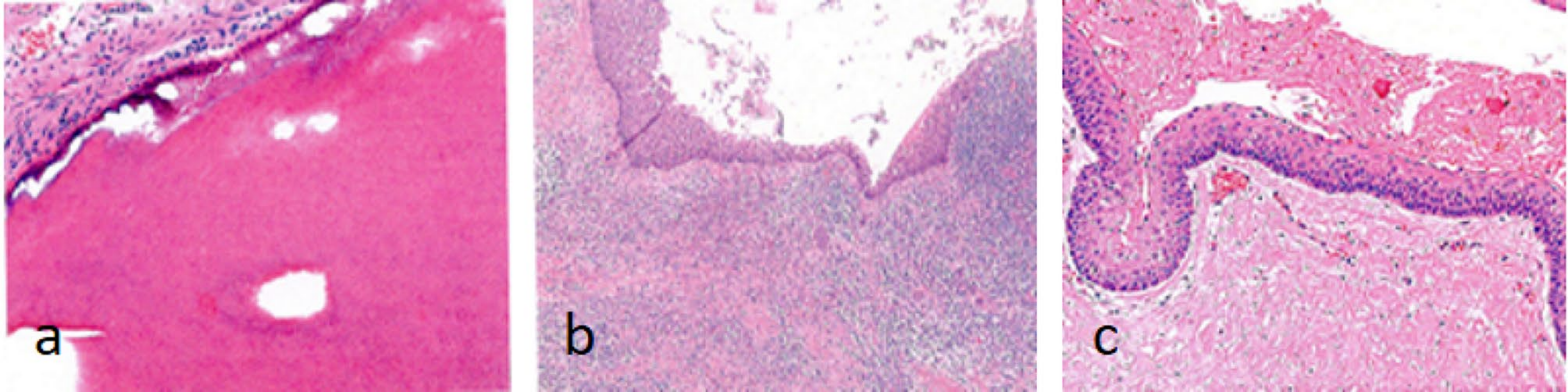
The histopathological examination images: (**a**) dentigerous cyst, (**b**) radicular cyst, (**c**) keratocyst.

All patients had some degree of rhinorrhea with postnasal drip after the operation, but it usually resolved within 2 weeks. CT showed that CMS was cured 8–12 weeks after surgery in group A and group B (Figs. 3, 4). The histopathological examination confirmed the diagnosis of OC (Fig. 5). No patients complained about nasal obstruction or facial paresthesia 36 months after surgery. No other major long-term complications were observed after 36 months of follow-up. No recurrence of OC in all patients observed 36 months follow-up.

## Discussion

OCs are lesions that destroy the jaw bone and develop from the proliferation and degeneration of the epithlium^7^. They account for 7–12% of biopsies among all oral and maxillofacial diagnoses^8,9^. RCs are the most common inflammatory maxillofacial cystic lesions that arise from infected and necrotic tooth pulp. RCs are the most common OC lesion, accounting for more than 50% of all OCs^2^. RCs appear to result from infection and necrosis in tooth pulp and account for more than 50% of all OCs^8–11^. Dentigerous cysts are the second most common cysts of the jaw, comprising 14–20% of all jaw cysts^12^. OCs can reach the maxillary sinus easily through areas of weakened bone^2^. The cyst and sinus membrane always adhere tightly because of bone resorption and inflammation.

Traditionally, the CDL procedure is performed in this situation to cure sinusitis. The standard CDL procedure is associated with more complications, including facial swelling, pain, mild temperature elevation and functional destruction of the sinus. Removal of the sinus packing postoperatively is always difficult for the patient.

Endoscopic surgery for OCs in the maxillary sinus has been reported in the literature^2,13^. However, in transnasal endoscopic surgeries, the cyst cannot be removed under direct vision. The approach has a narrow field of view that causes difficultly in the operation due to dead angles. This approach is not adequate for the removal of cyst membranes adhering to the tooth root or large intact cysts through the small orifice. Meanwhile the root apical resection and retrograde filling which is necessary in some OC surgery could not be performed by endoscopic surgery.

IMA has been performed to facilitate sinus irrigation postoperatively, and it has been reported that the IMA was closed in 82% of 367 cases 3 months postoperatively^14^. An analysis of the ciliary flow patterns in rabbits revealed that the nasal antral window did not cause redirection of the mucociliary clearance pattern^15^. In future work, we are preparing to take advantage of functional endoscopic sinus surgery (FESS) to insert the epidural catheter through the natural ostium of the sinus and evaluate the postoperative effect.

Our objective in this study was to evaluate the efficacy of postoperative sinus irrigation. The approach we report could relieve postoperative reactions, such as facial swelling and pain, reduce the probability of sinus infection and protect the function of the maxillary sinus.

## Conclusion

Sinus irrigation following large odontogenic cyst surgery involving the maxillary sinus can serve as an alternative to standard CDL and has the advantages of fewer complications, reduced trauma, restoration of the mucosa and more satisfactory results.
